# Role and therapeutic effects of skeletal muscle-derived non-myogenic cells in a rat myocardial infarction model

**DOI:** 10.1186/s13287-020-1582-5

**Published:** 2020-02-18

**Authors:** Hiroko Iseoka, Shigeru Miyagawa, Atsuhiro Saito, Akima Harada, Yoshiki Sawa

**Affiliations:** Department of Cardiovascular Surgery, Osaka University Graduate School of Medicine, Yamadaoka, 2-2, Suita, Osaka 565-0871 Japan

**Keywords:** Regenerative therapy, Stem cells, Cell markers, Heart failure

## Abstract

**Background:**

Transplantation of skeletal myoblast sheets is a promising strategy for the treatment of heart failure, and its therapeutic effects have already been proven in both animal disease models and clinical trials. Myoblast sheets reportedly demonstrate their therapeutic effects by producing many paracrine factors. Although the quality of processed cells for transplantation can be evaluated by the positive ratio of CD56, a myoblast marker, it is unclear which cell populations from isolated cells produce paracrine factors that have an impact on therapeutic effects, and whether these therapeutic effects are closely correlated with CD56-positive cells isolated from the skeletal muscle is also unclear. Therefore, we hypothesized that CD56-negative cells as well as CD56-positive cells isolated from the skeletal muscle produce paracrine factors and have therapeutic effects in skeletal muscle-derived cell sheet therapy for heart failure.

**Methods:**

Cell surface and intracellular markers of CD56-negative non-myogenic cells (NMCs) and CD56-positive myoblasts were evaluated. We also analyzed cytokine expression, tube formation ability, and stem cell mobilization in both cell populations. Finally, we assessed the therapeutic effects of the cell populations in a rat myocardial infarction model.

**Results:**

Analysis of cell surface and intracellular markers revealed that CD56-negative NMCs expressed fibroblast markers and a higher level of mesenchymal cell markers, such as CD49b and CD140a, than myoblasts. Both NMCs and myoblasts expressed various cytokines in vitro with different expression patterns. In addition, NMCs induced tube formation (control vs. myoblasts vs. NMCs: 100 ± 11.2 vs. 142 ± 8.3 vs. 198 ± 7.4%) and stem cell mobilization (control vs. myoblasts vs. NMCs: 100 ± 6.8 vs. 210 ± 22.9 vs. 351 ± 36.0%) to a higher degree in vitro than did myoblasts. The effect of NMCs and myoblasts on the improvement of cardiac function and suppression of myocardial fibrosis in rat myocardial infarction model was comparable.

**Conclusion:**

These results indicate that NMCs exhibit therapeutic effects in skeletal muscle-derived cell sheet therapy for heart failure. Thus, accurate parameters correlating with therapeutic effects need to be further explored.

## Background

Heart failure remains a major cause of death due to a limited regenerative capacity of the cardiac tissue. As a substitute for existing therapy, stem cell transplantations have been widely studied, and some stem cell therapies have already been applied clinically [1, 2]. Skeletal myoblasts, which are the progenitors responsible for the regeneration of the skeletal muscle, are a promising cell type with many clinical advantages such as easy expansion, lack of tumorigenic potential, and the possibility for use for autologous transplantation [3]. Previous studies have shown that sheet-shaped myoblasts can improve cardiac function in animal disease models as well as in clinical trials [4–6]. Further, the therapeutic effect of sheet-shaped myoblasts may be based on the production of factors that can induce angiogenesis or mobilization of stem cells, such as vascular endothelial growth factor (VEGF), hepatocyte growth factor (HGF), and stromal cell-derived factor-1 (SDF-1) [7–10].

In the clinical application of cell-based therapies, cell surface markers such as CD34 (a marker of hematopoietic stem cells), CD73, CD90, and CD105 (a marker of mesenchymal stem cells, MSCs), which may represent the targeting ability of cell tissue products, have been analyzed and have shown promise as stable and effective treatments for damaged tissues [11]. Although several markers, such as myogenin, MyoD, and desmin, have been reported as markers of myoblasts [12, 13], the cell surface marker CD56 has been thought to be more useful for evaluation of myoblast because of its simple method for measurement. However, the relationship between the ratio of CD56-positive cells and the therapeutic efficacy of skeletal muscle-derived cells is poorly understood. Moreover, little is known about the characteristics of skeletal muscle-derived CD56-negative non-myogenic cells (NMCs) and their potential therapeutic efficacy. Previous studies have demonstrated that several types of NMCs exist in the skeletal muscle, such as fibroblasts, endothelial cells, and mesenchymal cells [14, 15]. However, it is unclear whether these cells have a therapeutic effect on heart failure. Therefore, we hypothesized that NMCs as well as myoblasts isolated from the skeletal muscle produce paracrine factors and have therapeutic effects in skeletal muscle-derived cell sheet therapy for heart failure.

## Methods

### Cell culture

Human skeletal muscle-derived cells were isolated from the vastus medialis muscle of patients with heart failure using collagenase (Thermo Fisher Scientific, Waltham, MA, USA) and TrypLE Select (Thermo Fisher Scientific) and cultured in skeletal muscle growth medium (Lonza, Basel, Switzerland), as described previously [16]. The collection of the skeletal muscles from patients and isolation of skeletal muscle-derived cells were performed as part of treatment for heart failure by cell transplantation, with the surplus cells after treatment being used for this study. The patients had ischemic heart disease or dilated cardiomyopathy and were aged between 20 and 70 years.

Differentiation of myoblasts was induced by culturing in Dulbecco’s modified Eagle’s medium supplemented with 2% fetal bovine serum and 5nM insulin growth factor-1 (PeproTech, Rocky Hill, NJ, USA) for 5–7 days. Human MSCs (Lonza, PT-2501) and normal human dermal fibroblasts (Lonza) were cultured in MSC growth medium (Lonza) and fibroblast growth medium (Lonza), respectively, following the instruction protocol.

### Flow cytometry and cell sorting

Cultured cells were enzymatically dissociated with TrypLE select, washed, and labeled by fluorescence-conjugated primary antibodies for CD56 (1:20), CD31 (1:10), CD34 (1:10), CD90 (1:200), CD105 (1:10), CD49b (1:20), CD140a (1:20), and CD201 (1:5) (BD Biosciences, Franklin Lakes, NJ, USA) for 30 min at 4 °C and then assessed using the FACScanto II system (BD Biosciences). Next, cells were labeled with antibodies for desmin (1:25, DAKO, Santa Clara, CA, USA) and TE-7 (1:12, Merck Millipore, Burlington, MA, USA) after fixation using BD Cytofix Buffer (BD Biosciences), followed by incubation with fluorescence-conjugated secondary antibodies (Thermo Fischer Scientific). For screening of cell surface markers, human cell surface marker screening panel (BD Biosciences) was used following the manufacturer’s instructions.

Cultured cells were sorted into CD56-positive myoblasts and NMCs using FACSAria Sorter (BD Biosciences). The separated cells were cultured and then used for further analysis and transplantation.

Data were analyzed using Diva (BD Biosciences) or FlowJo software (TreeStar, Ashland, OR, USA).

### Cytokine measurement

Supernatants of cell sheets were collected and analyzed by commercially available fluorescence-dyed microsphere-based immunoassay (R&D Systems, Minneapolis, MN, USA) following the manufacture’s protocol. The concentrations of multiple cytokines/chemokines were measured using the Bio-Plex suspension array system (Bio-Rad, Hercules, CA, USA).

### Endothelial cell tube formation assay

Culture inserts (Corning, Corning, NY, USA) containing 1.95 × 10^5^ myoblasts or NMCs were placed above 1.25 × 10^5^ human umbilical vein endothelial cells (HUVECs) seeded on matrigel-coated cell culture plates. Positive control cultures consisted of inserts without cells supplemented with 10 ng/ml VEGF (R&D Systems). After culturing for 18 h, HUVECs were labeled by incubation with 5 μg/ml Calcein-AM (BD Biosciences) for 30 min at 37 °C. Vessel-like network structures were examined under fluorescence microscopy, and images were taken. Tube formation [total area (mm^2^)] was quantified using MetaMorph (Molecular Devices, San Jose, CA, USA).

### Migration assay

A migration assay was performed using FluoroBlok insert (BD Biosciences) containing 5 × 10^4^ MSCs placed above 1.0 × 10^6^ myoblasts seeded on cell culture plates. Control cultures comprised inserts placed over cell culture plates without cells supplemented with 150 ng/ml SDF-1 (R&D Systems). The FluoroBlok insert system allows the quantification of cells that have migrated through the insert by a bottom-reading fluorometer. After culturing for 6 h, MSCs were stained with 4 μg/ml Calcein-AM for 30 min at 37 °C, and fluorescence signals of the migrated cells were measured using a SH-9000 plate reader (COLONA ELECTRIC, Ibaraki, Japan).

### Transplantation of cell sheets into a chronic myocardial infarction (MI) rat model

All animal experiments and care were conducted humanely in compliance with the Guide for the Care and Use of Laboratory Animals published by the National Institutes of Health. The left anterior descending artery of athymic nude rats (F344/NJcl-rnu/rnu; CLEA Japan, Tokyo, Japan) was permanently ligated through left thoracotomy under general anesthesia administered via endotracheal intubation. After 2 weeks, rats underwent re-thoracotomy for the exposed pericardial space. Rats were randomly divided into a sham group or two cell-sheet-transplanted groups divided as per myoblasts or NMCs (*n* = 11 each). Myoblasts or NMCs were cultured on temperature-responsive culture dishes (CellSeed, Tokyo, Japan) for 1 day at 37 °C in the growth medium, and cell sheets were harvested by reducing the temperature to room temperature. Cell sheets were transplanted over the epicardium of the anterior and lateral left ventricular walls.

### Transthoracic echocardiography

Transthoracic echocardiography was performed using a SONOS 7500 machine (Philips Medical Systems, Eindhoven, Netherlands) and a 12-MHz annular-array transducer on rats under general anesthesia, which was administered without endotracheal intubation 2 weeks after the coronary artery ligation (just before transplantation) and 2 and 4 weeks after transplantation. The hearts were imaged in short-axis, two-dimensional views at the level of the papillary muscles, and the left ventricular end-systolic and -diastolic dimensions (LVESD and LVEDD, respectively) were determined. The left ventricular ejection fraction (LVEF) was calculated by the Pombo’s method [(LVEDD^3^ − LVESD^3^)/LVEDD^3^].

### Histological analysis and immunohistochemistry

The harvested heart was fixed with 4% paraformaldehyde, frozen in liquid nitrogen, and cryosectioned. Masson’s trichrome staining was performed to examine both myocardial fibrosis in the peri-infarct region and infarct size. Myocardial fibrosis was calculated as a percentage of the fibrotic area to the myocardial area, and the infarct size was calculated by the ratio of the infarcted portion of the endocardial circumference to the length of the entire endocardial circumference using the Metamorph software. To assess capillary density, sections were labeled with primary antibody for α-smooth muscle actin (αSMA; 1:100, DAKO) and fluorescence-conjugated isolectin-B4 (ILB-4), followed by incubation with fluorescence-conjugated secondary antibodies. For capillary density measurement, ILB-4-positive cells in 10 randomly selected fields in the peri-infarct area were counted and calculated as the number of positive cells/mm^2^ [17]. For measurement of ILB-4/αSMA double-positive vessel, αSMA- or ILB-4-positive cells were counted in the same way as described for capillary density, and the ratio of αSMA-positive cells in ILB-4-positive cells was calculated.

### Real-time polymerase chain reaction

Total RNA was extracted using an RNeasy fibrous tissue kit (Qiagen, Hilden, Germany), and cDNA was synthesized using an Omniscript RT kit (Qiagen). Real-time polymerase chain reaction (PCR) was performed using TaqMan PCR master mix on a 7500 fast real-time PCR system (Thermo Fisher Scientific). The following genes were analyzed using TaqMan gene expression assays (Thermo Fisher Scientific): *MMP2* (Rn01538170_m1), *MMP9* (Rn00579162_m1), *GAPDH* (Rn01775763_g1), *HGF* (Hs00300159_m1), *SDF-1* (Hs00930455_m1), and *VEGF* (Hs99999070_m1). Because human-specific genes were not detected in all rats, we calculated the detection rate of human-specific genes rather than performing comparative quantification. Relative gene expression was calculated using the ΔΔCt method.

### Statistical analysis

All statistical analyses were performed using GraphPad Prism version 6.07 (GraphPad Software, La Jolla, CA). Differences between groups were assessed by one-way analysis of variance (ANOVA). Comparisons among different time points and between experimental groups were performed with two-way ANOVA, followed by Tukey’s post hoc test.

## Results

### Phenotypic characteristics of NMCs

**Fig. 1:**
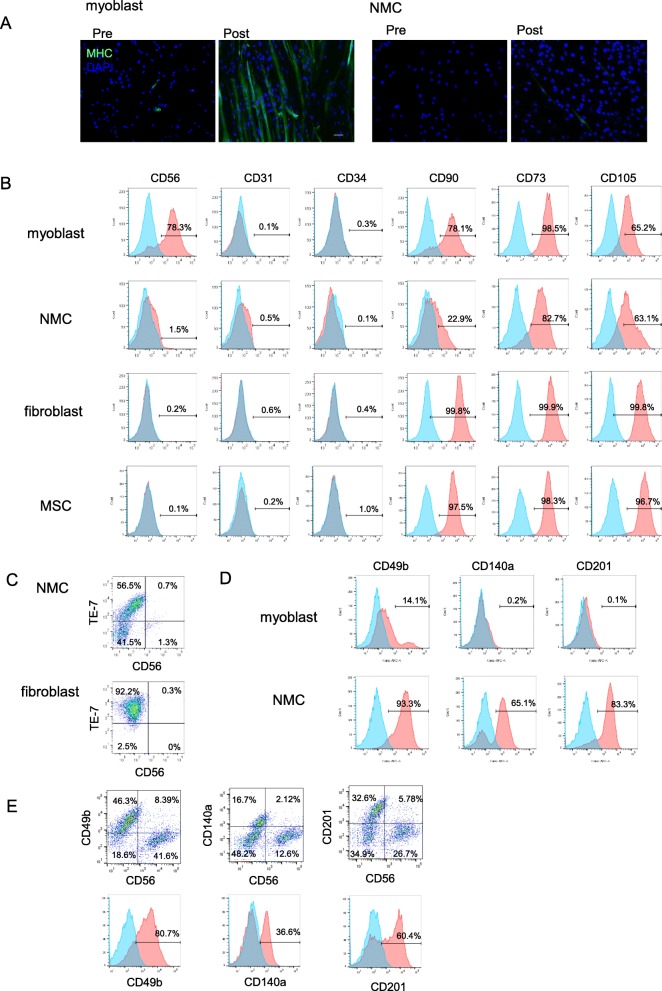
Characterization of NMCs. **a** Myogenic differentiation ability of NMCs and myoblasts was assessed by culturing cells under myogenic differentiation conditions and immunohistochemistry (scale bar 50 μm). **b**–**d** Expression of cell surface and intracellular markers in NMCs, myoblasts, fibroblasts, and MSCs was analyzed by flow cytometry. Isotype control is shown in blue, and the antibody-stained sample is shown in red. **e** Double staining of CD56 and cell surface markers highly expressed in NMCs. The histogram shows the positive ratio of each marker in the CD56-negative population. NMCs, non-myogenic cells; MSC, mesenchymal stem cell; *MHC*, Myosin heavy chain

To perform phenotypic characterization of CD56-negative NMCs, enzymatically digested and cultured muscle cells were divided into CD56-positive myoblasts and CD56-negative NMCs by cell sorting. First, to verify the myogenic capacity of CD56-negative NMCs and myoblasts, cells were cultured under myogenic differentiation conditions. Myoblasts formed myosin heavy chain-positive myotubes, whereas NMCs did not form myotubes (Fig. 1a). Next, the expression of cell surface and intracellular markers in NMCs was analyzed by flow cytometry. The expression of endothelial cell markers, CD31 and CD34, was low in NMCs (Fig. 1b). Furthermore, the expression of fibroblast markers, CD90 and TE-7, was lower in NMCs than in fibroblasts (Fig. 1c). These results show that not all NMCs were fibroblasts. Next, we performed a screening assay of cell surface markers to further reveal the markers of NMCs. We examined the expression of cell surface markers in myoblasts and NMCs by flow cytometry. CD49b, CD140a, and CD201 were found to be highly expressed in NMCs as compared with their expression in myoblasts (Fig. 1d). In addition, the double staining of CD56 and CD49b, CD140a, or CD201 in a mixed cell population of myoblasts and NMCs revealed that CD49b was the most highly expressed in NMCs among the three markers (Fig. 1e).

### Expression of cytokines

**Fig. 2:**
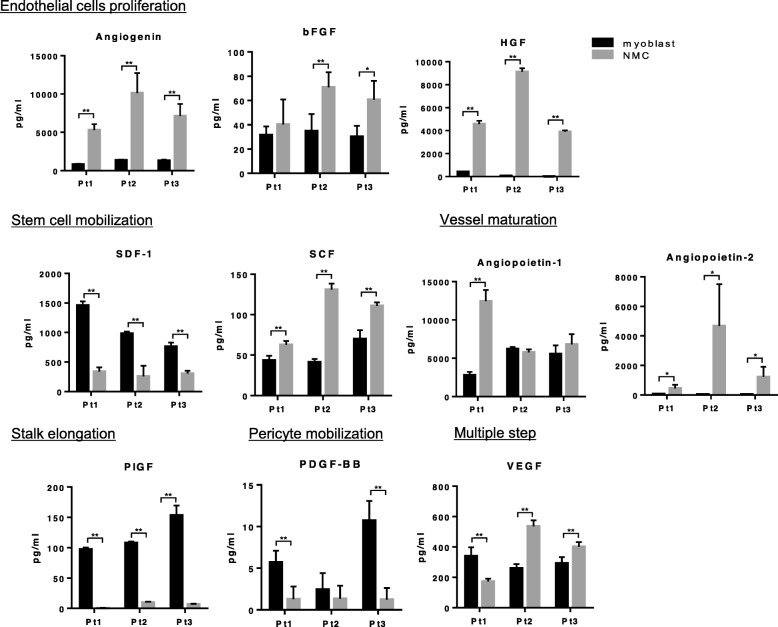
Expression of cytokines. In vitro cytokine production in myoblasts and NMCs was determined by fluorescence-dyed microsphere-based immunoassay. Myoblasts and NMCs from three patients were analyzed. Pt, patient; *p < 0.05, **p < 0.01

We analyzed the expression of cytokines in myoblasts and NMCs derived from three patients. The expression of SDF-1 (stem cell mobilization factor), placental growth factor (PIGF) (stalk elongation factor), and platelet-derived growth factor-BB (PDGF-BB) (pericyte mobilization factor) was significantly higher in myoblasts, whereas the expression of angiogenin, basic fibroblast growth factor, HGF (endothelial proliferation factors), and angiopoietin-1,2 (vessel maturation factors) was significantly higher in NMCs (Fig. 2). The difference in the expression of VEGF (the factor related to multiple angiogenesis processes) between the two cell types varied among patients.

### In vitro performance assay of paracrine factors

**Fig. 3:**
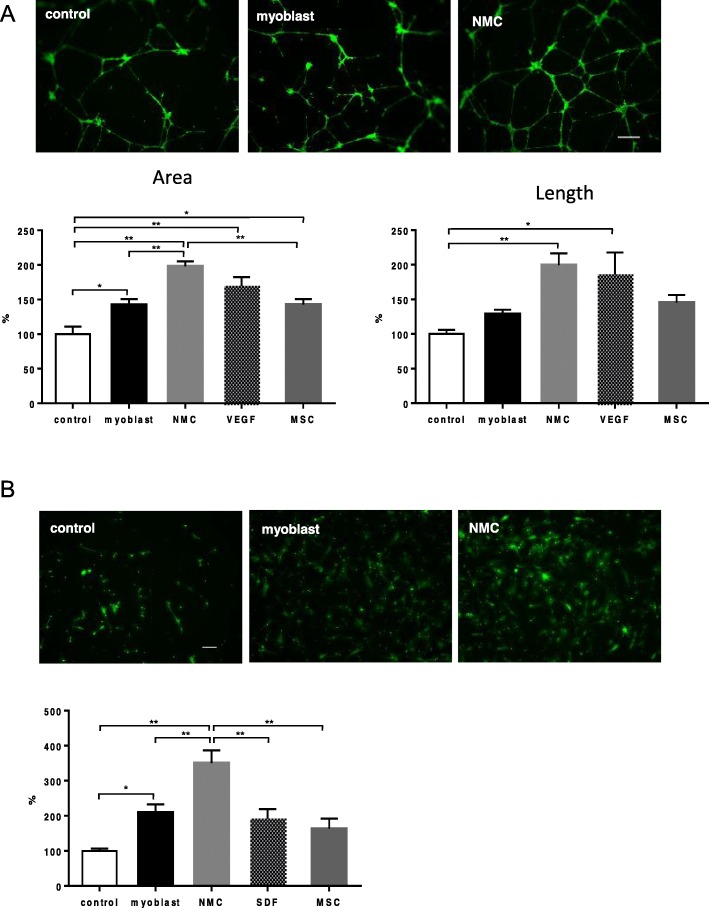
In vitro functional assay of paracrine factors. **a** Angiogenic properties were assessed by endothelial tube formation assays using HUVECs (scale bar 400 μm). Fluorescence images of tube structure formed by HUVECs and quantification data of the area of the tube structure (ratio to control) are shown. **b** Stem cell homing property was assessed by migration assay using MSCs (scale bar 100 μm). Fluorescence signals of the migrated cells, ratio to control, are indicated. HUVEC, human umbilical vein endothelial cells. *p < 0.05, **p < 0.01

To examine the angiogenic property of paracrine factors, an endothelial tube formation assay was performed using cell culture inserts and HUVECs. After culturing HUVECs with myoblasts or NMCs without direct contact, both groups formed significantly more vessel-like structures as compared with the medium only (Fig. 3a). In addition, NMCs formed significantly more vessel-like structures than myoblasts.

Next, we performed a migration assay using cell culture inserts and MSCs. After culturing MSCs with myoblast or NMCs without direct contact, the migrated MSCs were significantly increased in both cell groups (Fig. 3b). Furthermore, NMCs induced significantly higher migration of MSCs than myoblasts.

### Therapeutic effect

**Fig. 4:**
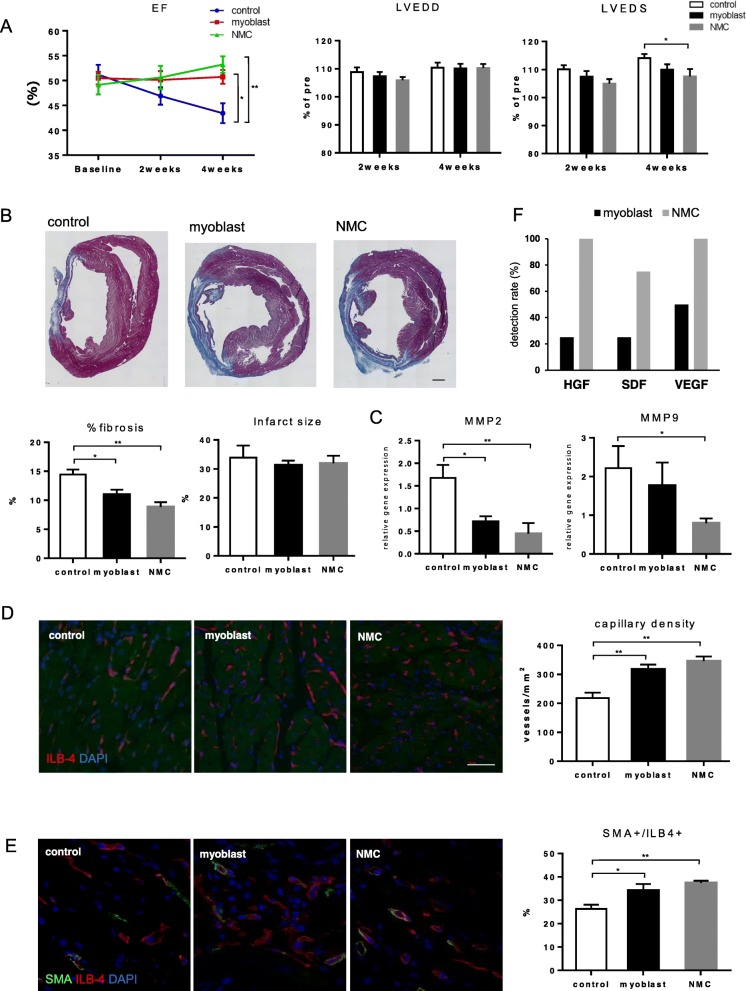
Therapeutic potential of NMC or myoblast cell-sheets. **a** Cell sheets consisting of myoblasts or NMCs were transplanted by left thoracotomy into nude rats subjected to left coronary artery ligation 2 weeks before transplantation. Sham rats served as controls. **a** LVEF, LVESD, and LVEDD were serially assessed by transthoracic echocardiography. Myocardial fibrosis (**b**), capillary density (**d**), and ratio of ILB-4/αSMA double-positive vessel in ILB-4 cells (**e**) in the peri-infarct area 4 weeks after cell-sheet transplantation were assessed by Masson’s trichrome staining and immunohistochemistry, respectively (scale bars: **b** 1000 μm, **d** 50 μm, **e** 20 μm). **c** Expression of fibrosis-related gene was assessed by real-time PCR. **f** Expression of human-specific gene in host myocardium was assessed by real-time PCR. LVEF, left-ventricular ejection fraction; LVESD, left-ventricular end-systolic dimension; LVEDV, left-ventricular end-diastolic dimension; ILB-4, isolectin-B4; αSMA, α-smooth muscle actin. *p < 0.05, **p < 0.01

To assess the therapeutic potential of myoblasts and NMCs, cell sheets consisting of myoblasts or NMCs were transplanted over the epicardial surface of chronic MI rat hearts, and cardiac function was evaluated by echocardiography before and at 2 and 4 weeks after cell sheet transplantation. LVEF was found to improve at 4 weeks post-transplantation, which gradually decreased in the control group over the 4-week period, although there was no significant difference between the myoblast and NMC groups (Fig. 4a). Furthermore, left ventricular end-systolic dimensions were significantly decreased in the NMC group as compared with those in the control group at 4 weeks after transplantation, whereas there was no significant difference between the myoblast and NMC groups. No significant difference was observed in left ventricular end-diastolic dimensions.

Moreover, transplantation of cell sheets consisting of myoblasts or NMCs showed a significant attenuation in myocardial fibrosis and expression of fibrosis-related genes, such as MMP2 and MMP9 as compared with the control group (Fig. 4b, c). There was no significant difference in infarct size of each group. In addition, transplantation of cell sheets consisting of myoblasts or NMCs significantly increased ILB-4-positive capillary and ILB4/αSMA double-positive vessels as compared with that in the control group (Fig. 4d, e). Finally, to analyze the engraftment of transplanted cells, the expression of human-specific genes was measured by real-time PCR. As a result, human-specific angiogenic factors, such as HGF, SDF-1, and VEGF, were detected more frequently in the NMC group than in the myoblast group (myoblast vs. NMCs, HGF: 25% vs. 100%, SDF-1: 25% vs. 75%, VEGF: 50% vs. 100%, respectively) (Fig. 4f).

## Discussion

In this study, to verify whether the analysis of CD56 expression is appropriate to evaluate the therapeutic capacity of skeletal muscle-derived cells, we analyzed and compared the characteristics and the function of skeletal muscle-derived CD56-negative NMCs with CD56-positive myoblasts both in vitro and in vivo. Both NMCs and myoblasts expressed angiogenic factors with different expression patterns. In addition, NMCs had significantly higher in vitro function (promotion of endothelial cell tube formation and mobilization of stem cells) than did myoblasts. Furthermore, transplantation of sheet-shaped NMCs or myoblasts exhibited more profound function recovery than did the control in a rat MI model.

Previous studies demonstrated that NMC populations such as TE-7-positive fibroblasts and CD34+ endothelial cells were present in the skeletal muscle tissue [14, 15]. However, NMCs isolated in this study exhibited lower expressions of these cell markers but expressed high levels of mesenchymal cell markers, such as CD140a, CD201, and CD49b, suggesting that the isolated NMCs are not endothelial cells or fibroblasts but mesenchymal cells. Recently, it was reported that CD201 is a novel marker of mesenchymal progenitor cells in the human skeletal muscle [18], and NMCs analyzed in this study may be similar to this cell population, although the detailed function of mesenchymal progenitor cells in the skeletal muscle has not been well analyzed. In addition, previous reports have demonstrated that CD140a and CD201 are markers of mesenchymal progenitors in the skeletal muscle [18, 19]. However, in this study, CD49b had a higher positive ratio in the CD56-negative population than did CD140a, suggesting that CD49b may be a novel marker for mesenchymal progenitor cells in the skeletal muscle.

Bone marrow-derived MSCs (BM-MSCs) are important for the formation of hematopoietic stem cell niche [20, 21] and are a promising cell source for regenerative medicine for heart failure [22, 23] or cerebral infarction [24, 25] because they express various paracrine factors such as VEGF and HGF. Although NMC markers are not completely consistent with those of BM-MSCs, NMCs may be mesenchymal cells that maintain stem cell characteristics in the skeletal muscle by expressing paracrine factors such as HGF and stem cell factor.

In this study, NMCs expressed angiogenic factors in vitro and promoted angiogenesis in vitro and in a rat MI model, suggesting that NMCs exhibited therapeutic effects based on the production of angiogenic paracrine factors and following angiogenesis. Though NMCs showed a higher production of angiogenic factors and promotion of tube formation in vitro than did myoblasts, improvement of cardiac function in a rat MI model was comparable between NMCs and myoblasts. In addition, several paracrine factors, such as PIGF, PDGF-BB, and SDF-1, were expressed at a higher degree in myoblasts than in NMCs. It is reported that the administration of PIGF improves cardiac function and decreases inflammatory cytokines in a rat myocardial infarction model [26], and PDGF-BB has an anti-apoptotic effect on cardiomyocytes [27]. These data suggest that myoblasts may exhibit therapeutic effects via mechanisms different from those of NMCs. The functional analysis supporting the angiogenic effects in vivo, such as a hemodynamic test, may reveal differences in the mechanisms between the two cell types. Furthermore, cell populations mixed with NMCs and myoblasts in the appropriate ratio may have a higher therapeutic effect by cross talk of both cell populations [17, 28]. The detection rate of transplanted cells 4 weeks after transplantation was better in the NMC group than in the myoblast group. However, further studies evaluating long-term engraftment and outcome are warranted to explore the difference in function in vivo.

Although NMCs and myoblasts expressed different cell markers, both showed a comparable therapeutic effect in a rat MI model, suggesting that analysis of a single-cell marker alone may not be enough for the evaluation of processed cells for transplantation. Since both NMCs and myoblasts expressed various cytokines, multiple cytokine measurements or in vitro functional assays may be useful to evaluate the processed cells. The difference in the expression of VEGF between NMCs and myoblasts differed among patients. Although there is no detailed information about the patients, it may be related to age and gender. It is necessary to accumulate data on the characteristics and therapeutic effects of transplanted cells to determine the parameters correlated with the therapeutic effect. This may also be useful for the development of a more efficient cell source.

## Conclusion

In conclusion, this study demonstrated that CD56-negative NMCs expressed angiogenic factors, exhibited angiogenic functions, and functioned in stem cell mobilization in vitro. NMCs highly expressed mesenchymal cell markers such as CD49b and CD140a. Furthermore, NMCs exhibited therapeutic effects in a rat MI model by mainly producing angiogenic cytokines, suggesting that analysis of the expression of CD56 alone is not the best to evaluate the true quality of cell tissue products. Further studies concerning detailed characteristics of NMCs may be needed to elucidate appropriate cell makers that represent the therapeutic ability in skeletal stem cell derivatives.

## Data Availability

The datasets used and/or analyzed during the current study are available from the corresponding author on reasonable request.

## Abbreviations

BM-MSCs: Bone marrow-derived mesenchymal stem cells
HGF: Hepatocyte growth factor
HUVEC: Human umbilical vein endothelial cells
ILB-4: Isolectin B4
LVEDD: Left ventricular end-diastolic dimensions
LVEF: Left ventricular ejection fraction
LVESD: Left ventricular end-systolic dimensions
MI: Myocardial infarction
MMP: Matrix metalloproteinase
MSC: Mesenchymal stem cells
NMCs: Non-myogenic cells
PCR: Polymerase chain reaction
PDGF-BB: Platelet derived growth factor-BB
PIGF: Placental growth factor
SDF-1: Stromal cell-derived factor-1
VEGF: Vascular endothelial growth factor
αSMA: α-Smooth muscle actin
